# Primary School Students’ Experiences using Minecraft Education during a National Project-Based Initiative: An Irish Study

**DOI:** 10.1007/s11528-023-00851-z

**Published:** 2023-05-10

**Authors:** Eadaoin J. Slattery, Deirdre Butler, Michael O’Leary, Kevin Marshall

**Affiliations:** 1 Centre for Assessment Research, Policy and Practice in Education, Institute of Education, Dublin City University, Dublin, Ireland; 2 School of STEM Innovation and Global Studies, Institute of Education, Dublin City University, Dublin, Ireland; 3 Microsoft Education Ireland, Dublin, Ireland

**Keywords:** Game-based learning, Minecraft, Project-based learning, Mixed-methods, School

## Abstract

Minecraft Education is a digital game-based learning platform that is thought to support the development of twenty-first century competencies and skills. The purpose of this study is to explore primary students’ experiences of using Minecraft Education during an innovative national project-based initiative. The initiative had two phases: 1) educational episodes for teachers and students on how to use the platform and 2) a national competition that required students to re-imagine a sustainable version of their community. We used a mixed-methods design with a sample of classes taking part in the initiative. First, third to sixth class students (*N* = 173) completed a survey that examined: 1) learning opportunities with Minecraft Education, 2) ease of use, 3) usefulness, and 4) enjoyment. Eight focus group interviews were then conducted with a subsample of sixth class students (*n* = 30). Students indicated that 1) there were good opportunities for learning with Minecraft Education, particularly for creativity and collaboration, 2) the platform was easy to use and useful and, 3) using Minecraft Education was enjoyable. Thematic analysis of the qualitative data identified five themes: ‘collaboration’, ‘opportunities for creativity’, ‘immersive learning environment’, ‘student engagement’, and ‘technology and digital skills’. This research highlights the value of innovative project-based learning activities with Minecraft Education for supporting student learning.

In today’s digital world, technology integration to support high quality learning experiences is essential for the twenty-first century model of education. Integrating technology into teaching and learning can support and enhance students’ learning and equip them with the digital skills necessary to participate in the ‘digital age’ (Fraillon et al., 2019; OECD, 2019). Educational systems and educators are increasingly being encouraged to find innovative means to incorporate new technologies meaningfully into classroom practice (European Commission et al., 2017). Digital game-based learning (DGBL) is one way to integrate technology meaningfully while simultaneously supporting learning objectives. Interest in DGBL rapidly increased during Covid-19, as it became an effective tool to facilitate remote learning (Lehane et al., 2021). However, not all digital games have the same impact on learning (Bavelier & Green, 2019; Dale & Shawn Green, 2017). Minecraft is one popular digital game that can support a range of learning experiences for diverse learners (Baek et al., 2020). Using a mixed-methods design, this paper explores students’ experiences of using Minecraft Education (ME) during a novel project-based initiative throughout the island of Ireland called *Ireland’s Future is MINE*. The project was aligned with the United Nations Sustainable Development Goals (or Global Goals; UN, 2015) to support the development of transformative competencies and twenty-first century skills as outlined in the OECD 2030 Learning Compass framework (OECD, 2019).

## Technology Integration

Technology integration refers to the use of educational technologies (e.g., devices, software, etc.) for teaching and learning purposes (Backfisch et al., 2021). Across educational systems worldwide, various initiatives and schemes have been launched to increase schools’ technological infrastructure. For example, in April 2022, the Department of Education and Skills in the Republic of Ireland announced a **€**210 million grant for school technological infrastructure from 2022–2027. While the use of digital technologies among teachers has increased in recent years, less than 50% of teachers report they frequently use technology for teaching and learning, with wide variation across countries (Fraillon et al., 2020). However, notably, an increase in the use of technology for teaching and learning does not necessarily translate into enhanced student learning (OECD, 2015). The effect of digital technologies on student learning is dependent on how technologies are used, and the types of technology-supported activities embedded into learning experiences (Antonietti et al., 2022). DGBL can be used to integrate technology effectively into the classroom while supporting students’ learning.

## DGBL and Minecraft Education

DGBL is the use of digital games in learning environments (Breien & Wasson, 2021; Prensky, 2001) and includes both off-the-shelf games and special purpose games. There is an abundance of digital games that claim to support learning (Tettegah et al., 2015). DGBL can support learning if the game chosen is the most suitable instructional method and enhances the overall learning experience (Lehane et al., 2021; Schrier, 2018). However, as games vary on many dimensions, different digital games are likely to generate different effects on learning. Minecraft is one game, which has unique opportunities for learning and supports several principles of key learning theories (Slattery et al., 2022). It is a sandbox game, which means it has no pre-determined goal. This gives players a great degree of freedom and creativity in terms of how they play the game. Players move around virtually infinite 3D worlds that consist of cubic blocks. The core gameplay involves using these blocks to build 3D structures. The versatile nature of the game as well as its popularity, led to a surge in the use of Minecraft for educational purposes (Callaghan, 2016).

This interest resulted in the release of ME, which is very popular with educators. As of October 2021, 35 million teachers and students across 115 countries were licenced to use ME (Minecraft, 2021). The educational version is a special purpose DGBL platform designed specifically for use in the classroom. The gameplay is similar to the off-the-shelf version, but many features make it easy to use for teachers. For example, students can use the code builder to create in their ME world; teachers can monitor students’ work, adjust some settings, provide feedback and track students’ progress with features like the Book & Quill tool. As a versatile DGBL platform, educators can easily modify ME to meet the needs of their students and their learning objectives.

## Transformative Competencies for the 21^st^ Century

The OECD 2030 Learning Compass Framework highlights the importance of developing students’ transformative competencies (OECD, 2019). Transformative competencies are a set of holistic concepts, which include the acquisition and application of knowledge, skills, attitudes, and values that students need to succeed in the world (OECD, 2019). The framework specifies three core competencies: 1) creating new value (ability to come up with novel innovations), 2) reconciling tensions and dilemmas (ability to work through challenging or conflicting circumstances), and 3) taking responsibility (ability to understand actions have consequences; for detailed review, see OECD, 2019). These competencies can be learned by incorporating them into the curriculum (OECD, 2019). Key twenty-first century skills such as collaboration, creativity, critical thinking and problem-solving skills underlie the development of transformative competencies. Digital technologies such as DGBL can be integrated into learning environments to help students develop these competencies and skills (European Commission et al., 2017) and ME can meaningfully support the development of these key skills and competencies across a range of learners (Lehane et al., 2021).

## Developing Transformative Competencies using Minecraft Education

Project-based learning is a key approach for the development of student competencies and skills (Birdman et al., 2021). Project-based learning involves two core components: 1) a posed challenge that the learning activity is centred around, and 2) a final product that addresses the posed challenge (Blumenfeld et al., 1991). Project-based learning with Minecraft has been implemented across a range of learning areas. For example, in 2016, the Mindrising Games was launched to involve young people in the Irish State’s 100-year commemoration of the 1916 Easter Rising (a catalyst for the formation of the Irish State). Students used Minecraft to create their own virtual worlds, which explored the events of the Rising and imagined the future of Ireland over the next 100 years (Butler et al., 2016). The project was aligned with key curricular skills from the Irish, History and English curriculum.^1^ Moreover, in a narrative review of the literature, Lehane et al. (2021) found that Minecraft can be used to facilitate project-based learning activities and these activities can support student learning. Therefore, project-based learning using Minecraft may be one approach to enhance student learning.

## Ireland’s Future is MINE: Project-Based Learning using Minecraft Education

In 2021, Ireland’s Future is MINE, a novel national initiative was launched across the island of Ireland involving the use of ME. It was a project-based initiative in partnership between Dream Space™ at Microsoft Ireland^2^ and the national broadcaster (RTÉ), which was aligned with the United Nations Sustainable Development Goals, particularly the goal of ‘Sustainable Cities and Communities’ (UN, 2015), to support the development of transformative competencies and twenty-first century skills in primary school students. Schools were able to register for free ME accounts. The initiative had two phases. In the first phase, six educational episodes aired on RTÉ.^3^ The episodes were designed to introduce teachers and students to ME and how to use it. In each episode, students were given a challenge (e.g., build a Mars Rover) and had to complete it using ME. The second phase of the initiative involved a competition for students to design a sustainable version of their local community or area in ME and make a video displaying their work.^4^ The project incorporated many key curricular areas in the Irish primary school curriculum. This study follows on from this initiative and explores students’ experiences of using ME during the project with a particular focus on the development of twenty-first century skills, namely, collaboration, creativity, and problem solving. The study addresses the following research questions:

1. What are students’ experiences of using ME for project-based learning activities designed to develop their twenty-first century skills and competencies, namely collaboration, creativity, and problem solving?
2. How do students rate the learning opportunities, usefulness, ease of use and enjoyment of using ME in a school setting?

## Methods

This research study had two components. The first component involved the administration of student questionnaires. The questionnaire contained four instruments designed for the current study: (1) learning opportunities with ME, (2) ease of use, (3) usefulness, and (4) enjoyment. Teachers administered hardcopy questionnaires to students during class time at the end of the initiative. In the second phase, we conducted semi-structured focus groups with students to examine in-depth their experiences of using ME during Ireland’s Future is MINE. The focus groups were also conducted at the end of the initiative. The university’s research ethics committee granted ethical approval for the research.

### Participants

Teachers taking part in the Ireland’s Future is MINE initiative were invited to register their classes’ interest in participating in the research associated with the initiative. Advertisements were circulated on social media and teachers were sent an invitation when they signed up for the project. Informed parental consent and participant assent were obtained from all participants before taking part in the research. In total, eight schools (3^rd^ to 6^th^ class) completed the student questionnaire (*N* = 173, 95 boys). Students ranged in age from 8 to 13 years (*M* = 10.65, *SD* = 1.37). Out of these eight classes, three sixth class groups took part in the student focus groups (*N* = 30, 16 boys and 14 girls).

### Measures

Four scales were used (1) learning opportunities with ME, (2) ease of use, (3) usefulness, and (4) enjoyment. These scales were chosen to understand students’ experiences of using the platform. All instruments are presented in Appendix A.

#### Perceived Learning Opportunities

Bourgonjon et al. (2010) developed the learning opportunities scale to assess the extent to which an individual perceives that using digital games in educational settings can offer them learning opportunities. We adapted the original measure to make it relevant for the current study (i.e., learning opportunities for the development of twenty-first century skills with ME). The scale comprised eight items. Students rated each item on a 5-point scale ranging from 1 (*strongly disagree*) to 5 (*strongly agree*). Examples of items include ‘Using Minecraft allows me to work with other students’, ‘Using Minecraft gives me a chance to solve problems,’ and ‘Using Minecraft helps me to come up with ideas’. Items were averaged to create an overall score. Higher scores indicated higher levels of opportunities for learning with ME. Reliability analysis indicated good internal consistency (Cronbach’s α = 0.815).

#### Technology Acceptance

We adapted the technology acceptance scale (Chu et al., 2010; Hwang et al., 2013) for the current study. This scale assesses students’ perceptions of the use of digital games for learning. The technology acceptance scale has two subscales, which assess 1) perceived usefulness and 2) perceived ease of use. Both scales had four items rated on a 5-point scale ranging from 1 (*strongly disagree*) to 5 (*strongly agree*). Examples of items on the perceived usefulness scale include ‘Minecraft makes learning better’ and ‘Learning with Minecraft is more useful than other ways of learning’. Examples of items on the perceived ease of use scale include ‘I quickly learned how to play the game’ and ‘It was difficult for me to learn how to play Minecraft’. Items were averaged to create an overall score for each scale. Higher scores indicate higher levels of perceived usefulness / ease of use. Reliability analysis indicated adequate internal consistency (perceived usefulness Cronbach’s α = 0.678 and perceived ease of use Cronbach’s α = 0.620).

#### Enjoyment

A six-item scale was used to assess students’ enjoyment using ME. This scale was adapted from previous DGBL research (Giannakos, 2013; Venkatesh et al., 2002). Students rated the extent to which they enjoyed using ME (e.g., ‘Learning is more interesting using Minecraft’ and ‘Using Minecraft is fun’) on a 5-point scale ranging from 1 (*strongly disagree*) to 5 (*strongly agree*). Items were averaged to compute an overall score. In the current study, the scale had good internal consistency reliability (Cronbach’s α = 0.783).

## Focus Groups

The focus group interviews were semi-structured. We designed the schedule to encourage students to talk about their experiences of using ME in class with a focus on collaboration, creativity and problem solving. Prompts were included to encourage further conversation if required. The schedule was piloted a priori. Appendix B presents the focus group questions and prompts. The focus group commenced with a discussion of students’ class projects in order to develop rapport and help students to feel comfortable. Focus groups were conducted by the first author in schools during February and March 2022. The audio was recorded. Focus group duration ranged from 11:29 to 32:40 min (*M* = 20:02). Recordings were transcribed verbatim by the research team.

### Reflexivity

Reflexivity is the process of reflecting on how you as a person (e.g., your values and attitudes) and your methodological choices during the research process influence the production of knowledge (Braun & Clarke, 2021). Reflexivity is a core part of high-quality qualitative research. The first author kept a reflective diary for the duration of the project to reflect on their thoughts and practices.

### Data Analysis

Results from the three scales (learning opportunities, technology acceptance and enjoyment) are presented descriptively. Exploratory analyses were conducted to examine differences between class groups on the variables and associations between the variables. We then used reflective thematic analysis to analyse the data, as we were interested in shared patterns of meaning across participants. The analysis was carried out in accordance with Braun and Clarke’s (2006) framework for thematic analysis: 1) familiarisation, 2) initial coding, 3) theme search, 4) theme review, 5) theme definition and, 6) write up. The first author familiarised themselves with the transcripts, and coded and recoded the data using a combined inductive and deductive approach. The codes were then grouped under preliminary themes, which were reviewed and refined.

## Results and Discussion

Descriptive statistics are first presented followed by the results from the thematic analysis.

### Descriptive Statistics

Descriptive statistics for the learning opportunities, technology acceptance (usefulness and ease of use) and enjoyment scales for each class group are shown in Table 1. Each scale used a 5-point Likert scale ranging from 1 (*strongly disagree*) to 5 (*strongly agree*). As can be seen, the scores across all class groups were above the neutral midpoint of the scale (i.e., 3), indicating that on average students felt there were good learning opportunities with ME, ME was useful for learning and easy to use, and students enjoyed using ME. Notably, the average scores for the ease-of-use scale and the enjoyment scale were above 4, suggesting a very positive response.

**Table 1:** Descriptive Statistics

	*n*	*Min*	*Max*	*M*	*SD*
3^rd^ Class					
Learning Opportunities	41	2.13	5	3.81	.56
Usefulness	40	2.50	5	3.95	.69
Ease of Use	40	2.50	5	4.43	.60
Enjoyment	40	2.50	5	4.49	.54
4^th^ Class					
Learning Opportunities	25	2.75	5	4.10	.62
TA Usefulness	27	2.75	5	4.07	.55
TA Ease of Use	27	3.50	5	4.51	.51
Enjoyment	27	3.50	5	4.69	.45
5^th^ Class					
Learning Opportunities	24	2.50	5	3.76	.60
Usefulness	24	2.00	5	3.74	.87
Ease of Use	24	3.25	5	4.36	.56
Enjoyment	24	3.67	5	4.63	.41
6^th^ Class					
Learning Opportunities	79	2.38	5	3.69	.57
Usefulness	79	2.25	5	3.57	.68
Ease of Use	79	2.25	5	4.30	.63
Enjoyment	80	3.33	5	4.52	.53

*TA* Technology Acceptance

Responses to each item on the learning opportunities with ME are provided in Table 2. There was strong evidence that the majority of students regarded ME as a tool for collaboration and creativity. For example, 87.5% to 96.3% of students across class groups indicated that using ME allowed them to work with other students, and 93.7% to 100% of students indicated that ME helps them to be creative.

**Table 2:** Responses to Items on the Learning Opportunities with Minecraft Scale

	Strongly disagree %	Disagree %	Neither agree nor disagree %	Agree %	Strongly agree %
3^rd^ Class					
Using Minecraft allows me to work with other students	0	2.4	7.3	53.7	36.6
Using Minecraft gives me the chance to solve problems	7.3	9.8	46.3	24.4	12.2
Using Minecraft helps me to be creative	0	0	2.4	17.1	80.5
Using Minecraft helps me to come up with new ideas	0	0	4.9	41.5	53.7
Using Minecraft allows me to discover new ways to solve problems	7.3	9.8	29.3	39	14.6
Using Minecraft gives me a chance to use knowledge learned in class	7.3	4.9	34.1	31.7	22
Using Minecraft helps me to better understand things we learn in class	7.3	14.6	26.8	39	12.2
Using Minecraft gives me a chance to use knowledge learned outside of class	14.6	12.2	24.4	17.1	31.7
4^th^ Class					
Using Minecraft allows me to work with other students	0	3.7	0	37	59.3
Using Minecraft gives me the chance to solve problems	0	15.4	15.4	50	19.2
Using Minecraft helps me to be creative	0	0	0	22.2	77.8
Using Minecraft helps me to come up with new ideas	0	0	15.4	23.1	61.5
Using Minecraft allows me to discover new ways to solve problems	3.8	11.5	19.2	46.2	19.2
Using Minecraft gives me a chance to use knowledge learned in class	0	11.1	14.8	48.1	25.9
Using Minecraft helps me to better understand things we learn in class	0	11.1	22.2	40.7	25.9
Using Minecraft gives me a chance to use knowledge learned outside of class	0	7.4	18.5	40.7	33.3
5^th^ Class					
Using Minecraft allows me to work with other students	0	0	12.5	66.7	20.8
Using Minecraft gives me the chance to solve problems	0	20.8	45.8	20.8	12.5
Using Minecraft helps me to be creative	0	0	4.2	20.8	75
Using Minecraft helps me to come up with new ideas	0	4.2	4.2	33.3	58.3
Using Minecraft allows me to discover new ways to solve problems	0	20.8	33.3	25	20.8
Using Minecraft gives me a chance to use knowledge learned in class	0	12.5	50	29.2	8.3
Using Minecraft helps me to better understand things we learn in class	4.2	41.7	20.8	25	8.3
Using Minecraft gives me a chance to use knowledge learned outside of class	0	4.2	20.8	58.3	16.7
6^th^ Class					
Using Minecraft allows me to work with other students	0	0	6.3	46.3	47.5
Using Minecraft gives me the chance to solve problems	1.3	7.5	50	32.5	8.8
Using Minecraft helps me to be creative	0	0	6.3	28.7	65
Using Minecraft helps me to come up with new ideas	1.3	1.3	11.3	50	36.3
Using Minecraft allows me to discover new ways to solve problems	0	21.3	50	20	8.8
Using Minecraft gives me a chance to use knowledge learned in class	2.5	13.9	38	35.4	10.1
Using Minecraft helps me to better understand things we learn in class	5.1	35.4	35.4	20.3	3.8
Using Minecraft gives me a chance to use knowledge learned outside of class	0	24.1	17.7	43	15.2

### Exploratory Analysis

To explore the quantitative data in more detail, we ran a series of exploratory analyses examining relationships between the variables and differences across classes.

#### Differences between Classes

In order to determine whether there were differences between classes on our measures (perceived learning opportunities, usefulness, ease of use and enjoyment), we ran a series of one-way ANOVAs. Statistically significant differences between classes were identified for perceived learning opportunities (*F*(3, 165) = 3.22, *p* = 0.024) and usefulness (*F*(3, 166) = 4.85, *p* = 0.003). No other statistically significant differences were found (all *p*s > 0.05). Follow up post-hoc Bonferroni tests indicated that fourth class students (*M* = 4.10, *SD* = 0.62) rated learning opportunities significantly higher compared to those in sixth class (*M* = 3.69, *SD* = 0.57; *p* = 0.014, *d* = 0.71). While students in third class (*M* = 3.95, *SD* = 0.69; *p* = 0.031, *d* = 0.56) and fourth class (*M* = 4.07, *SD* = 0.55; *p* = 0.008, *d* = 0.78) reported significantly higher usefulness compared to those in the sixth class (*M* = 3.57, *SD* = 0.68). These exploratory results suggest that there may be some differences between younger and older classes in their perceived learning opportunities associated with the platform and perceived usefulness of using ME. However, these results should be interpreted with caution, as there were low numbers in some class groups. Furthermore, the ratings by different class groups were aligned more often than not. However, it may be helpful for individuals who are designing similar initiatives and project-based learning experiences with ME in the future to keep these potential differences in mind.

#### Correlations between Variables

Table 3 displays Pearson correlations. As some class groups had a small sample size, correlations are reported for the sample as a whole. Correlations for each class group can be found in Appendix C. Both perceived learning opportunities (*r* = 0.686, *p* < 0.001) and enjoyment (*r* = 0.640, *p* < 0.001) showed positive strong correlations with usefulness. Learning opportunities had a moderate-to-strong correlation with enjoyment (*r* = 0.423, *p* < 0.001), while ease of use had a weak-to-moderate correlation with enjoyment (*r* = 0.283, *p* < 0.001). This pattern of correlation suggests that students who enjoyed the platform more also found it more useful, easier to use, and reported higher learning opportunities.

**Table 3:** Pearson Correlations

Variables	1.	2.	3.	4.
1. Learning Opportunities	-	.686***	.120	.423***
2. Usefulness		-	.131	.640***
3. Ease of Use			-	.283***
4. Enjoyment				-

*** *p* < .001

### Thematic Analysis

**Fig. 1:**
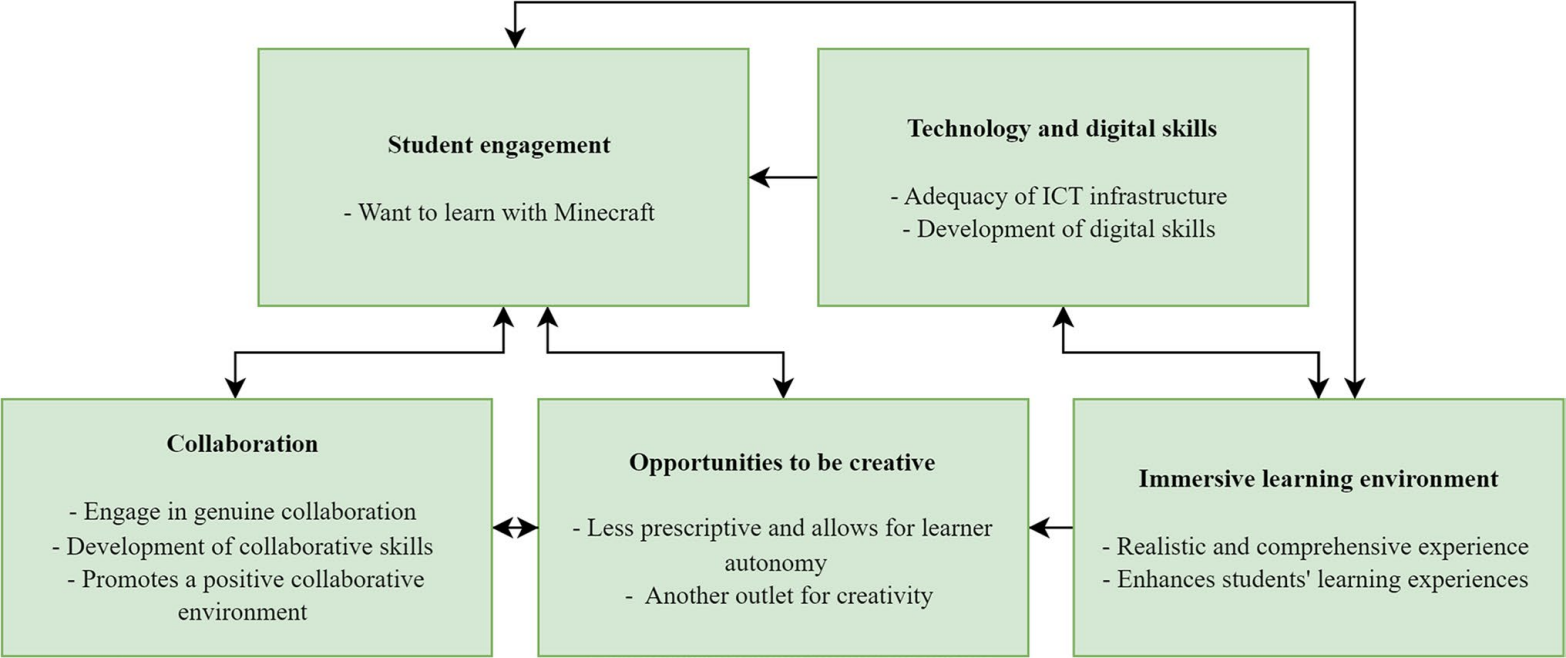
Thematic Framework

Five themes were identified (‘collaboration’, ‘opportunities to be creative’, ‘immersive learning environment’, ‘student engagement’, ‘technology and digital literacy’) from the analysis of the focus group interviews. The final thematic framework and relationship between themes is shown in Fig. 1.

#### Theme 1: Collaboration

The majority of students remarked how collaboration was a key part of their learning experience with ME, ‘It [ME] helps everyone work together’ (FG 1, Mary). Students were engaged in genuine collaboration while working on their projects. Genuine collaboration in the classroom occurs when students work interdependently, undertake a joint activity, and pool their knowledge, skills, and efforts (Evans, 2020). Students worked together towards a collective goal, ‘We all like, work together, and it is really cool because the end product we all worked together [on it] and put a lot of work into it.’ (FG 8, Oisin). Importantly, group members combined their knowledge and skills in pursuit of the group’s goal. Many students referenced how they learned from other students throughout the process, ‘You could work together more. You learn things from each other’ (FG 1, John) / ‘You have other people who already know how to do it and if you do not know how to do it, then there are people to help you.’ (FG 4, Simone). In the classroom, there are three types of student interaction: 1) promotive interaction, 2) oppositional interaction and 3) no interaction (Johnson & Johnson, 2002). Promotive interaction promotes true collaboration and occurs when members of a group support other members to complete tasks in pursuit of the group’s shared goal (Johnson & Johnson, 2002). A key feature of this type of interaction is group members explaining and modelling how to overcome a problem or sharing knowledge with others (Johnson & Johnson, 2002). This occurred frequently when students were using ME as evidenced by the above quotes, which provides further evidence that ME supports genuine collaboration.

Students got the opportunity to develop their collaboration skills. They planned and made group decisions on how best to manage and complete tasks. This involved deciding as a group how to assign roles, ‘We gave each other roles.’ (FG 2, Christopher). Moreover, students learned to overcome problems as a group and developed their conflict resolution skills. One student, Robin, described how they learned to respectfully communicate with one another if an issue arose, ‘[ME / this project] showed us that if someone had an opinion to maybe think about their opinion, and to communicate more, and if there is a problem, then not to start shouting, to try and communicate with them more.’ (FG 4). Students learned to listen to others’ opinions and ideas and adapt their own thinking in order to benefit the group:

I am not a very good person when it comes to listening to other people’s opinions. Once I have my opinion, I sort of stick to it. And I’m like ‘no… you have to do that’. It’s my opinion… I think it’s the best idea here. But when we did Minecraft I sort of had to listen to other people’s opinions and then I realised they worked really well and not just to always listen to my own opinion. (FG 4, Simone).

Notably, students highlighted how using ME helped them get along better with their classmates, ‘We got on with each other better’ (FG 4, Simone). There seemed to be less barriers to working together with ME than with other approaches used in the classroom, ‘It’s bringing the class together a bit more. Like, normally it is like sometimes the boys would go off and play, then the girls would. But I think it kind of brought us together more’ (FG 4, Robin). Overall, using ME supported genuine collaboration, improved students’ collaboration skills and promoted a positive collaborative environment.

#### Theme 2: Opportunities to be Creative

Students highlighted how using ME supported their creative process. That is, students regard it as a space that sparked their imagination and ideas, ‘You can be more creative with all the things you make’ (FG 2, Laura). Notably, students reiterated that they felt freer to be creative using ME. This is likely due to the freedom and choice available to students because of its open-ended nature in addition to the availability of the resources to create (i.e., there are more resources available to students in ME than in the physical world to represent their ideas and ignite their imagination). Game-based approaches with a degree of learner autonomy have been shown to support students’ creative skills (Davies et al., 2013). Ben articulates this in the following extract:

The fact that it is a sandbox means you can, just go in there and make it. You don’t have to do anything and you can just be creative and that way it’s really good… That is one of the reasons it’s a really good thing. (FG 6)

Throughout the focus groups, students reiterated how they felt there was no wrong answer in ME. They viewed using ME as less prescriptive with more opportunities for individual responses. This is a key factor, which can support the development of creativity in the classroom (Davies et al., 2013). When using ME students felt freer to come up with suggestions and try new things. For example, in the following extract Ann describes how she felt less constrained when using ME compared to other learning experiences in the classroom:

When you are doing Irish or Maths, there is like a right or wrong answer, but in Minecraft there isn’t really, so you can just do what you want… There is no real thing you can do wrong or anything like that. (FG 1).

Relatedly, the consequences of a mistake were seen as minimal. Mistakes are part of any learning process. In educational settings, mistakes are typically not desirable; however, environments that support learning through trial and error have many educational benefits (Weinzimmer & Esken, 2017). Students highlighted how they could learn from their mistakes in ME, ‘If you made a mistake, you could be able to know where you went wrong’ (FG 8, Dara). One student described how making a mistake could lead to other ideas:

When you make a mistake sometimes, it gives you an idea. Like when you are trying to make a table and you accidentally make something else you could just like… You could do this instead of making a table. (FG3, Sam).

Lastly, students identified how ME can give students who may not excel in traditional forms of creativity the opportunity to demonstrate their creative skills. One student described how ME enabled them to create something as they saw it in their mind:

It’s really good because you’re able to see it as you wanted it to be. I’m terrible at drawing so like I try, whenever I tried to draw something out it didn’t look good. But then if you can build it in Minecraft you can see it in 3D as well. (FG 6, Amy).

In summary, ME supports the creative process and fosters a creative environment in the classroom.

#### Theme 3: Immersive Learning Experience

The majority of students identified immersion as a key feature of using ME. Immersion refers to the perception that one is part of a true to life experience while participating in a digital experience (Dede, 2009). Students felt they were inside the game, ‘You’re kind of inside the project, and it’s much cooler.’ (FG 5, Ronan). Game-based learning approaches offer different degrees of digital immersion, which depend on the characteristics of the digital game (e.g., sensory, action, and symbolic characteristics; Dede, 2009). ME uses both action and symbolic features to achieve a sense of immersion. For example, students are able to interact with their builds via their avatar. Students referenced the similarities between the 3D digital world in ME and the real world, ‘If you build something in Minecraft, you can build it in real life. So, if you build something in Minecraft, then you can do it in real life.’ (FG 5, Andrew). This provides further evidence that students perceive they are participating in a realistic experience when using ME. Interestingly, students compared immersive learning with ME to traditional learning approaches typically used in the classroom. ‘At school you need to work it out and copy, but in Minecraft you can walk about and figure it out as you go.’ (FG 1, John).

Students did not realise they were learning when using the platform. ‘It doesn’t feel like work at all, it’s just like you are having a good time.’ (FG 2, Laura). In this segment, Laura seems to be referring to a sense of flow while working with Minecraft. Flow, an extreme degree of immersion, refers to a state of mind characterised by heighted absorption, concentration and enjoyment when completing an activity (Hamari et al., 2016; Hsu & Cheng, 2021). This is evidenced by Ronan, ‘I work better when I am playing Minecraft. I would concentrate more.’ (FG 5). Notably, immersion has been shown to enhance the learning experience and learning processes (Hsu & Cheng, 2021).

#### Theme 4: Student Engagement

This theme is related to the concept of flow. The view that using ME was an enjoyable and interesting learning activity was reiterated across students. ‘It is fun, it is enjoyable, and it helps you learn.’ (FG 8, Dara). This is notable, as previous research has shown that student engagement in DGBL has a positive effect on their learning (Hamari et al., 2016). Students want to learn with ME. ‘Everybody is interested in it and wants to do it.’ (FG 7, Jane). This is linked to student agency, which is a key aspect of the OECD 2030 Learning Compass framework. Student agency involves students becoming active participants in their learning and making decisions about what and how they will learn (OECD, 2019). One student described how they are more motivated to learn with ME (i.e., the how of learning):

People want to do it. Like for example, if there is a subject a lot of people might not like, like Irish, but since they do not like it, they don’t give it a shot, so they might not be good at it. So, then they dislike it even more, but with Minecraft, it is fun and you would actually want to learn while doing it (FG 6, Ben).

This extract highlights how students’ interest in ME can positively influence their learning. This is important as students’ academic engagement and motivation decreases as they progress through primary school (Gallup, 2014). These factors are linked to a variety of educational outcomes such as academic achievement (e.g., Bae et al., 2020). As such, educators are encouraged to find new ways to increase students’ academic engagement and motivation (Rowell & Hong, 2013). Overall, this theme captures how ME can be used in the classroom setting to increase academic motivation and engagement.

#### Theme 5: Technology Infrastructure and Digital Skills

Students identified barriers to using ME in the classroom in terms of technology infrastructure, particularly device infrastructure (i.e., old devices, not enough devices and restricted device use). Although technology accessibility is improving in Irish classrooms, technology infrastructure such as WiFi is still an issue in many schools (Department of Education and Skills, 2022). For example, in the below extract, Ann, describes how their devices were not fit for purpose:

Our laptops are a bit old. They are slow because they are quite old. We are starting to replace them now… sometimes it [the laptop] just randomly kicked you out. Sometimes it would take us 20 minutes to get into the world. And even if we had an hour, then we’d only have half an hour to actually do things, because it would take us a half hour to get in so it wasn’t the best, the laptops. (FG 1)

Two barriers influence the integration of technology into the classroom: first-order barriers (external factors) and second-order barriers (internal factors; Ertmer, 1999). Lack of adequate technology infrastructure is a first order barrier that must be overcome to achieve technological integration and advance students’ digital skills.

Students reiterated how their digital skills improved during the project. That is, their ability to use digital devices improved. Many students remarked how they initially found it challenging to adjust to playing ME on a computer/laptop:

It was hard getting used to, like, the binds [controls] because most of us were playing on the controller so it would be… hard to play with it on the mouse or just like that and the keyboard. Yeah so it was pretty hard to adjust to it. (FG 1, Evan)

The ability to use different digital devices proficiently is a key digital skill. While students found it difficult to adjust at the start, with use students became proficient at using it on a laptop/computer and found it easy to use, ‘But then we learned it [ME] and it was easier, so much easier.’ (FG 5, Ronan).

## Overall Discussion

This study used a mixed-methods design to examine students’ experiences of using ME during an innovative project-based initiative. The initiative had two phases: 1) a series of educational episodes focused on ME and 2) a national competition based on the United Nations’ Sustainable Development Goals (United Nations, 2015), which required students to work collaboratively to re-imagine a sustainable version of their community in ME. Quantitative data indicated that students felt that 1) there were good opportunities for learning with ME, particularly opportunities for creativity and collaboration, 2) ME was easy to use and useful and, 3) using ME was enjoyable. Exploratory analysis of this data suggested that students who enjoyed ME more found it more useful, easier to use and reported higher learning opportunities. Some differences were identified between younger and older classes in their perceived learning opportunities associated with the platform and perceived usefulness of using ME. However, as noted, these results should be interpreted with caution due to low numbers in some class groups. Thematic analysis of the qualitative data identified five themes: ‘collaboration’, ‘opportunities for creativity’, ‘immersive learning environment’, ‘student engagement’, and ‘technology infrastructure and digital skills’. Overall, the findings highlight the value of project-based learning initiatives with ME to support student learning.

### Limitations and Future Research

This study provides novel insights into students’ experiences of a project-based learning initiative with ME. However, the study has some limitations. First, we may have introduced selection bias by recruiting a convenience sample for the survey and focus groups. It is possible that the experiences of students in these classes would have differed from a randomly recruited sample of classes taking part in the initiative. Second, we only administered student questionnaires after the initiative, which limits our ability to ascertain whether students’ scores improved over the course of the initiative. A pre-test post-test design would have been a stronger study design; however, this was not possible as the initiative began at the start of the school year with restricted time to obtain parental consent for pre-test assessments. Additionally, we did not obtain participant feedback on our thematic analysis. Participant feedback can strengthen qualitative research (King & Brooks, 2017); however, this was not possible due to the young age of our sample.

## Conclusion

Educational systems, educational practitioners and policy makers should consider novel ways to incorporate project-based learning experiences with ME into educational settings. First, project-based learning experiences using ME can facilitate the development of key twenty-first century skills and competencies. Second, learning with ME provides students with a realistic and comprehensive learning experience, which brings their learning to life and enhances their overall learning experience. Third, students want to learn with ME and student agency (students becoming active participants in their own learning) is a fundamental principle of teaching and learning in the twenty-first century model of education. However, issues with technology infrastructure must be addressed in order to realise the full potential of the platform as an educational tool.

## Data Availability

Data not available due to ethical restrictions. The participants of this study did not give written consent for their data to be shared publicly.

## Appendix A

Tables

**Table 4:** Learning Opportunities with Minecraft Scale

	Strongly disagree	Disagree	Neither agree nor disagree	Agree	Strongly
1. Using Minecraft allows me to work with other students					
2. Using Minecraft gives me a chance to solve problems					
3. Using Minecraft helps me to be creative					
4. Using Minecraft helps me to come up with new ideas					
5. Using Minecraft allows me to discover new ways to solve a problem					
6. Using Minecraft gives me a chance to use knowledge learned in class					
7. Using Minecraft helps me to better understand things we learn in class					
8. Using Minecraft gives me a chance to use knowledge learned outside of class					

4,

**Table 5:** Technology Acceptance Scale

	Strongly disagree	Disagree	Neither agree nor disagree	Agree	Strongly
**Ease of Use Subscale**					
1. It was difficult for me to learn how to play Minecraft					
2. I quickly learned how to play the game					
3. It was not difficult for me to use the game during learning activities in school					
4. Minecraft was easy to learn and use					
**Usefulness Subscale**					
1. Minecraft makes learning better					
2. Minecraft helps me to gain new knowledge					
3. Minecraft helps me to get useful information when I need it					
4. Learning with Minecraft is more useful than other ways of learning					

5,

**Table 6:** Enjoyment Scale

	Strongly disagree	Disagree	Neither agree nor disagree	Agree	Strongly
1. Learning is more interesting using Minecraft					
2. Using Minecraft is fun					
3. I enjoy learning using Minecraft					
4. Learning with Minecraft is exciting					
5. I hope there will be other chances to use Minecraft for learning					
6. I would recommend Minecraft to other students for learning					

6

## Appendix B

Tables

**Table 7:** Questions and Prompts used in the Focus Groups with Students

General Questions	General Prompts
Can you tell me about your Ireland’s Future is MINE project?	
What did you learn when you were working on your Ireland’s Future is MINE project?	• *Tell me more about that*
	• *How did you learn that?*
What was it like to work with your classmates using Minecraft?	• *Did you work with each other in groups?*
	• *How did you divide the work?*
	• *Were there any difficulties you encountered when working together?*
	• *Did Minecraft help you to work better with your classmates? Why/why not?*
	• *Tell me about a time when you worked together using Minecraft*
Do you think Minecraft helps you to be creative?	• *Why/why not?*
	• *Tell me about something creative you did using Minecraft*
Did you encounter any problems when you were working on your project?	• *Tell me about the problem(s)*
	• *How did you solve these problems?*
	• *What did you do about it?*
	• *What helped you to solve problems?*
Did you face any challenges when working with Minecraft?	• *Tell me about the challenges*
	• *What did you do about it?*
	• *How did you overcome the challenges?*
What are some of the good things about using Minecraft for a school project?	• *What are the best things about using Minecraft?*
	• *What did you like most about Minecraft for a school project?*
	• *Was there anything you liked about using Minecraft for a school project?*
Would you like to use Minecraft for school projects in the future?	• *Why?*
What advice would you have for a teacher who wanted to bring Minecraft education to their school?	

7

## Appendix C

Table

**Table 8:** Pearson Correlations

	1	2	3	4
3^rd^ Class				
1. Learning Opportunities	-	.500**	-.135	.402**
2. Usefulness		-	–.352*	.601***
3. Ease of Use			-	.034
4. Enjoyment				-
4^th^ Class				
1. Learning Opportunities	-	.834 ***	.605**	.514 **
2. Usefulness		-	.662***	.692 ***
3. Ease of Use			-	.611 **
4. Enjoyment				-
5^th^ Class				
1. Learning Opportunities	-	.763 ***	-.187	.416*
2. Usefulness		-	-.159	.668 ***
3. Ease of Use			-	-.173
4. Enjoyment				-
6^th^ Class				
1. Learning Opportunities	-	.698***	.140	.397***
2. Usefulness		-	.302**	.691***
3. Ease of Use			-	.413***
4. Enjoyment				-

^*^ *p* < .01, ** *p* < .01, *** *p* < .001

^8^

## Footnotes

1 Sample projects can viewed here https://www.mindrising.ie

2 “Dream Space at Microsoft Ireland provides immersive, research-based STEM experiences for students, teachers and communities to enhance their STEM skills alongside crucial social-emotional skills such as empathy, creativity, ethical decision making and collaboration.” (Microsoft, 2023).

3 Episodes can be accessed here https://www.rte.ie/learn/irelands-future-is-mine/

4 Over 120 class projects were submitted. The highest ranked projects can be viewed here https://www.rte.ie/learn/irelands-future-is-mine/2022/0309/1285398-provincial-winners/
